# Supplementary figures and images for: Gut microbiota analyses of Saudi populations for type 2 diabetes-related phenotypes reveals significant association
Source: BMC Microbiol. 2022 Dec 13;22:301. doi: 10.1186/s12866-022-02714-8 (PMC9746012; doi:10.1186/s12866-022-02714-8)

**Fig. S1** Data processing and analysis pipeline for Saudi T2D 16S microbiota study

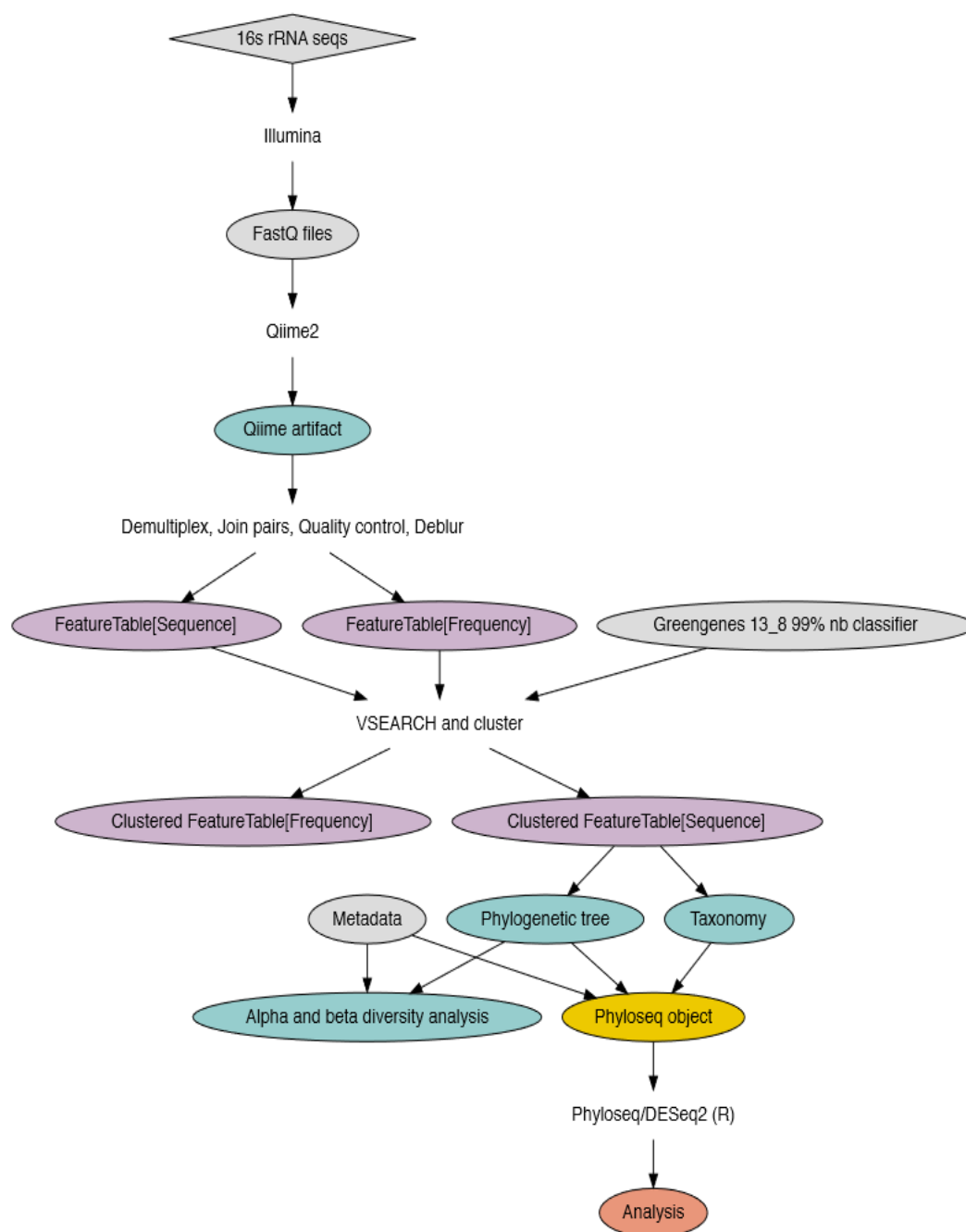

Supplement: Supplementary file 1 — Additional file 1: Figure S1. Data processing and analysis pipeline for Saudi T2D 16S microbiota study. [file 12866_2022_2714_MOESM1_ESM.pdf]

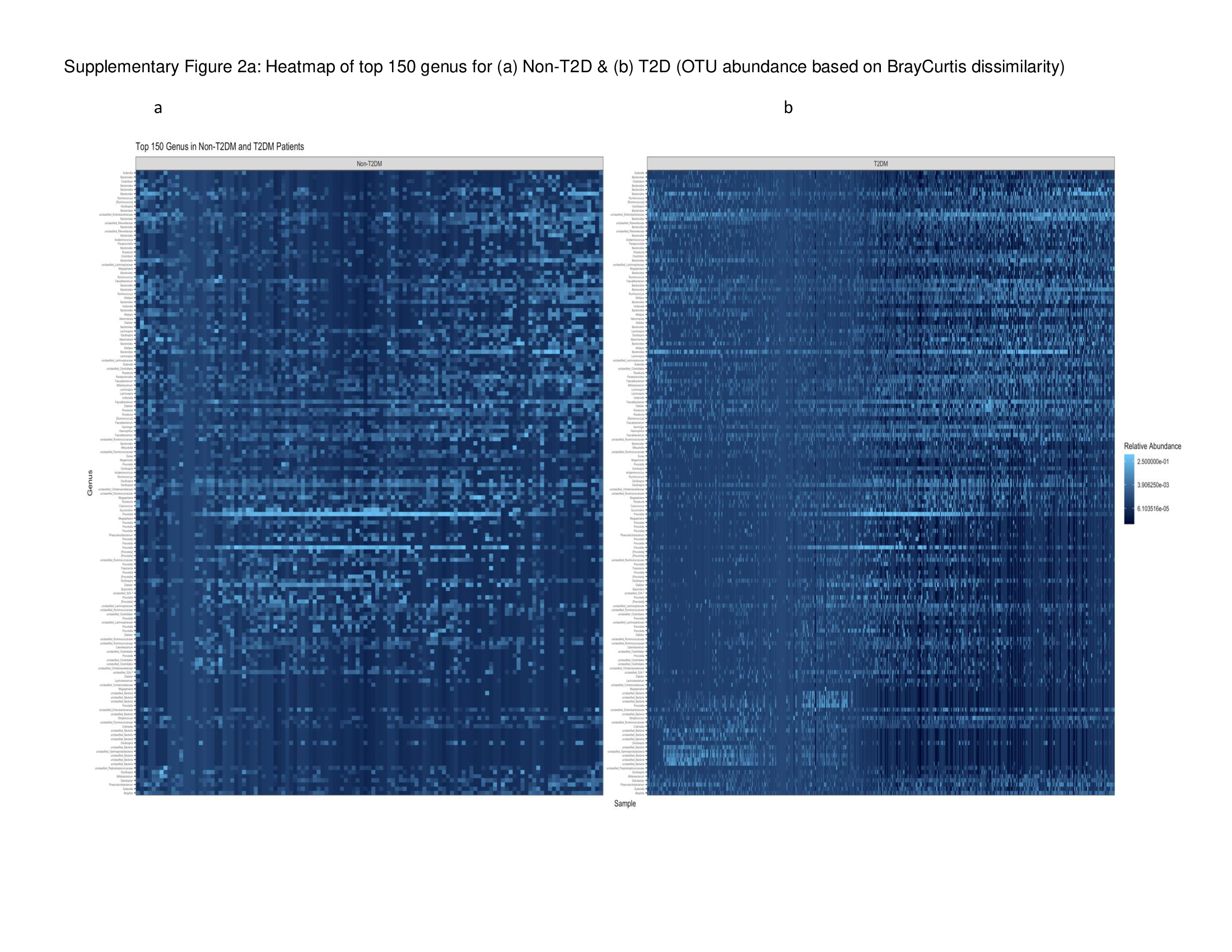

Supplement: Supplementary file 2 — Additional file 2: Supplementary Figure 2. a: Heatmap of top 150 genus for (a) Non-T2D & (b) T2D (OTU abundance based on BrayCurtis dissimilarity). b: Heatmap of top 50 genus for (a) Non-T2D & (b) T2D individuals listed respectively. [file 12866_2022_2714_MOESM2_ESM.zip › Figure-S2a.tiff]

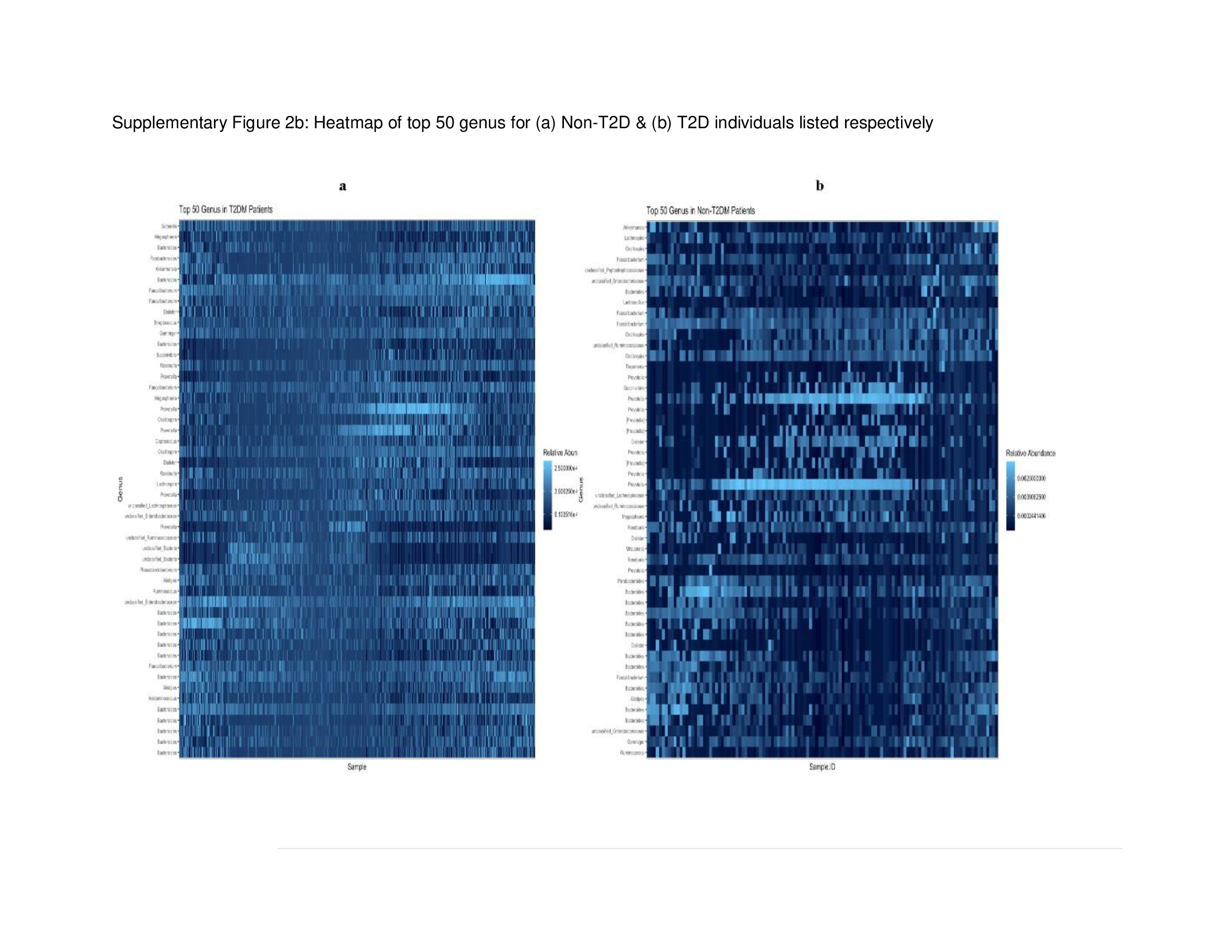

Supplement: Supplementary file 2 — Additional file 2: Supplementary Figure 2. a: Heatmap of top 150 genus for (a) Non-T2D & (b) T2D (OTU abundance based on BrayCurtis dissimilarity). b: Heatmap of top 50 genus for (a) Non-T2D & (b) T2D individuals listed respectively. [file 12866_2022_2714_MOESM2_ESM.zip › Figure-S2b.tiff]

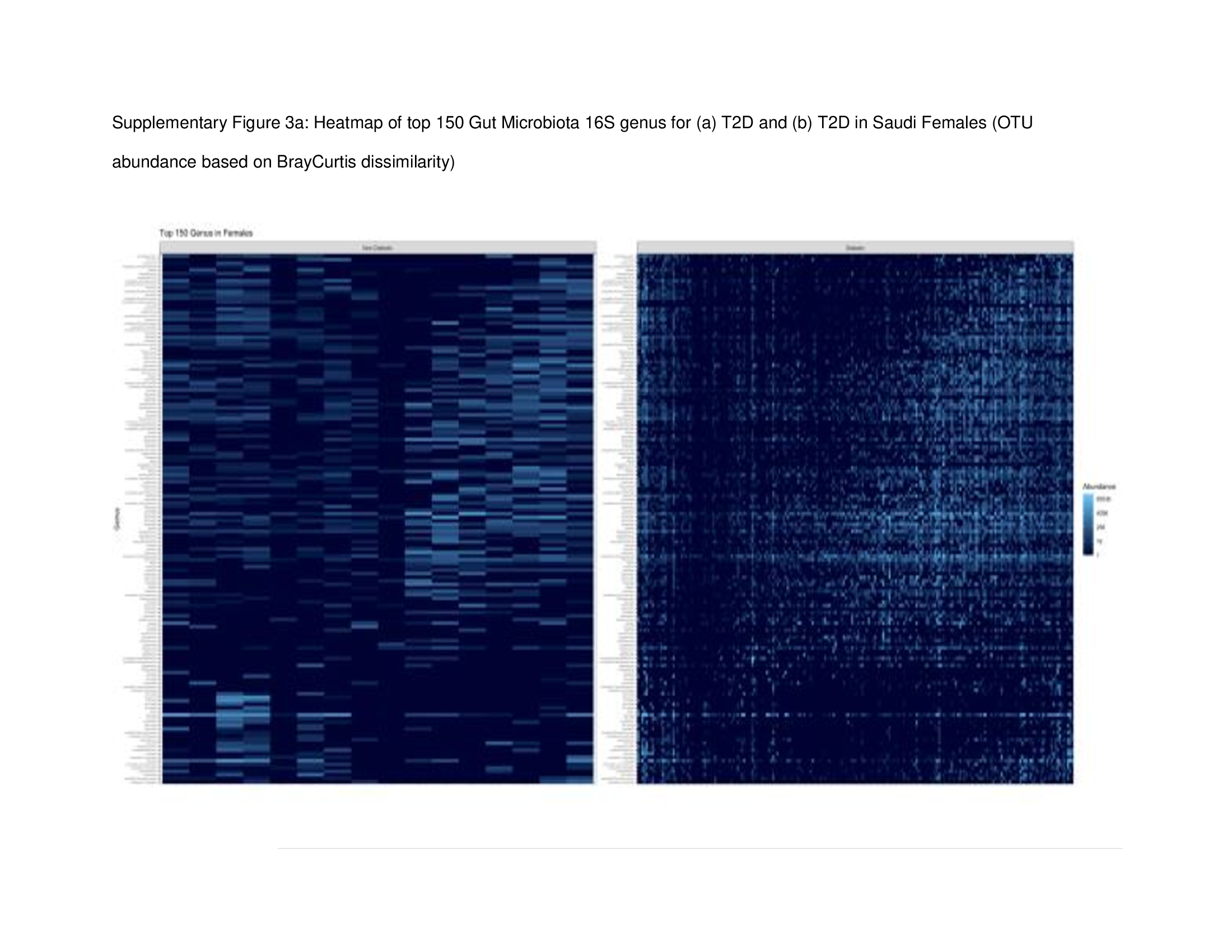

Supplement: Supplementary file 3 — Additional file 3: Supplementary Figure 3. a: Heatmap of top 150 Gut Microbiota 16S genus for (a) T2D and (b) T2D in Saudi Females (OTU abundance based on BrayCurtis dissimilarity). b: Abundance of Gut Microbiota 16S Taxonomic Composition of: a) non-T2D vs (b) T2D in Saudi Females. [file 12866_2022_2714_MOESM3_ESM.zip › Figure-S3a.tiff]

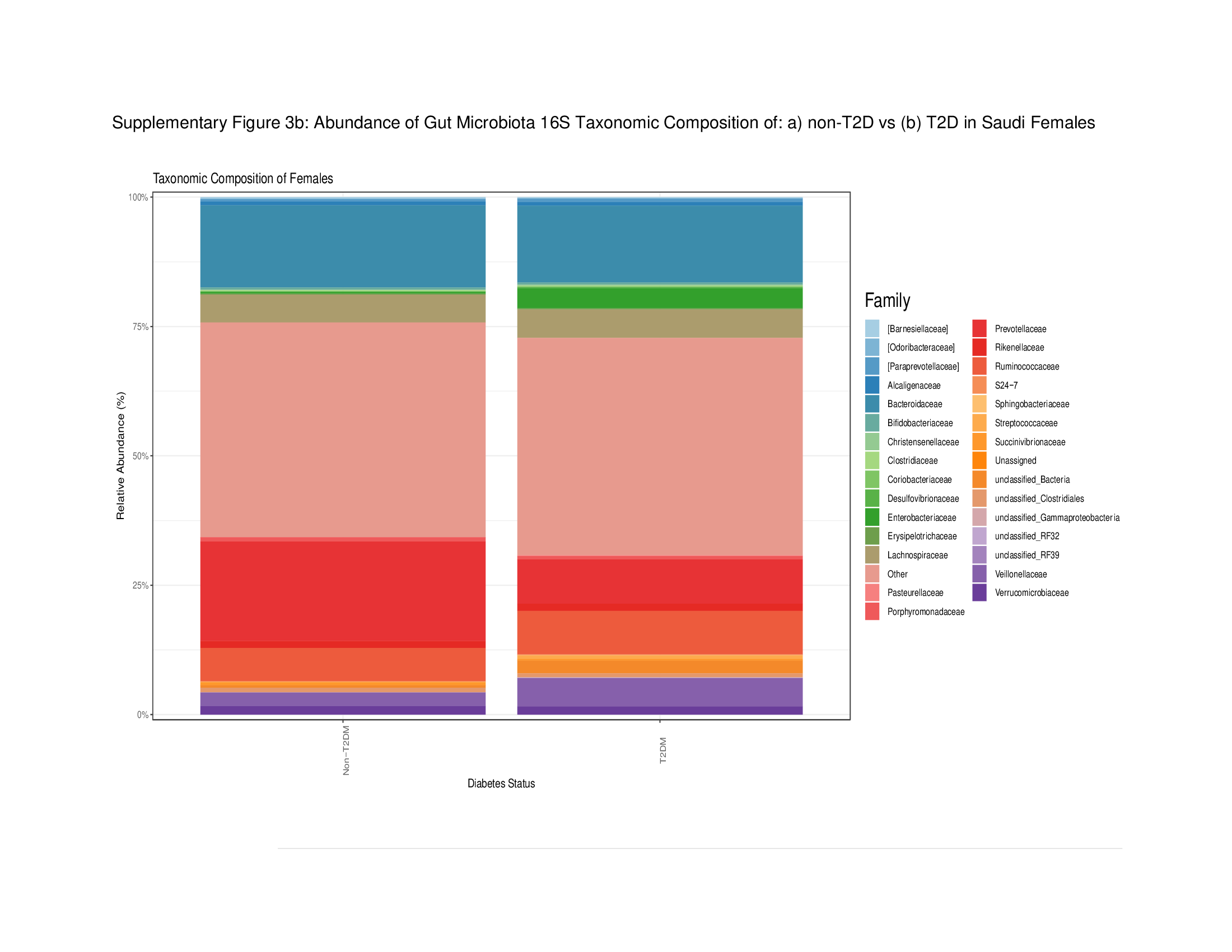

Supplement: Supplementary file 3 — Additional file 3: Supplementary Figure 3. a: Heatmap of top 150 Gut Microbiota 16S genus for (a) T2D and (b) T2D in Saudi Females (OTU abundance based on BrayCurtis dissimilarity). b: Abundance of Gut Microbiota 16S Taxonomic Composition of: a) non-T2D vs (b) T2D in Saudi Females. [file 12866_2022_2714_MOESM3_ESM.zip › Figure-S3b.tiff]

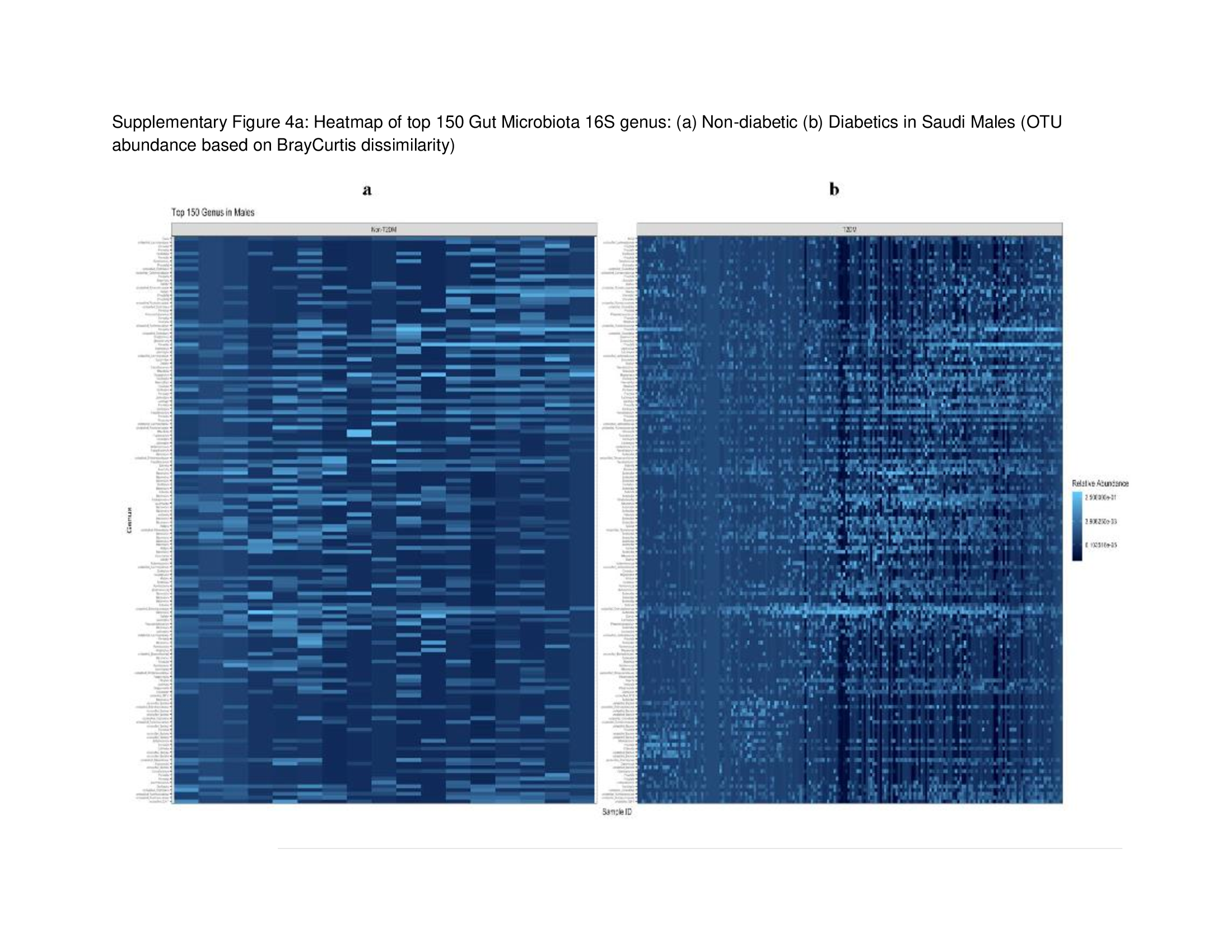

Supplement: Supplementary file 4 — Additional file 4: Supplementary Figure 4. a: Heatmap of top 150 Gut Microbiota 16S genus: (a) Non-diabetic (b) Diabetics in Saudi Males (OTU abundance based on BrayCurtis dissimilarity). b: Abundance of Gut Microbiota 16S Taxonomic Composition of: a) Non-T2D vs (b) T2D in Saudi Males. [file 12866_2022_2714_MOESM4_ESM.zip › Figure-S4a.tiff]

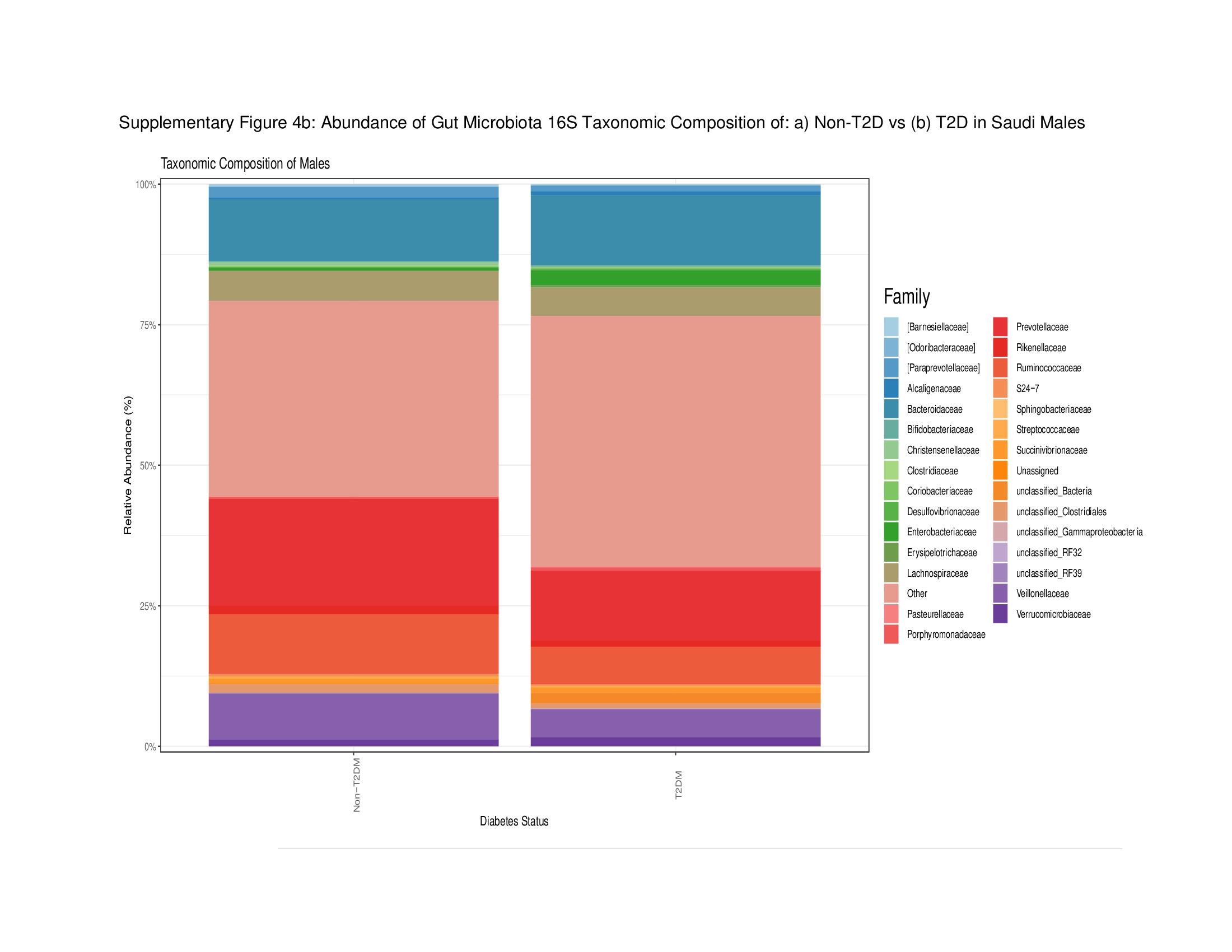

Supplement: Supplementary file 4 — Additional file 4: Supplementary Figure 4. a: Heatmap of top 150 Gut Microbiota 16S genus: (a) Non-diabetic (b) Diabetics in Saudi Males (OTU abundance based on BrayCurtis dissimilarity). b: Abundance of Gut Microbiota 16S Taxonomic Composition of: a) Non-T2D vs (b) T2D in Saudi Males. [file 12866_2022_2714_MOESM4_ESM.zip › Figure-S4b.tiff]

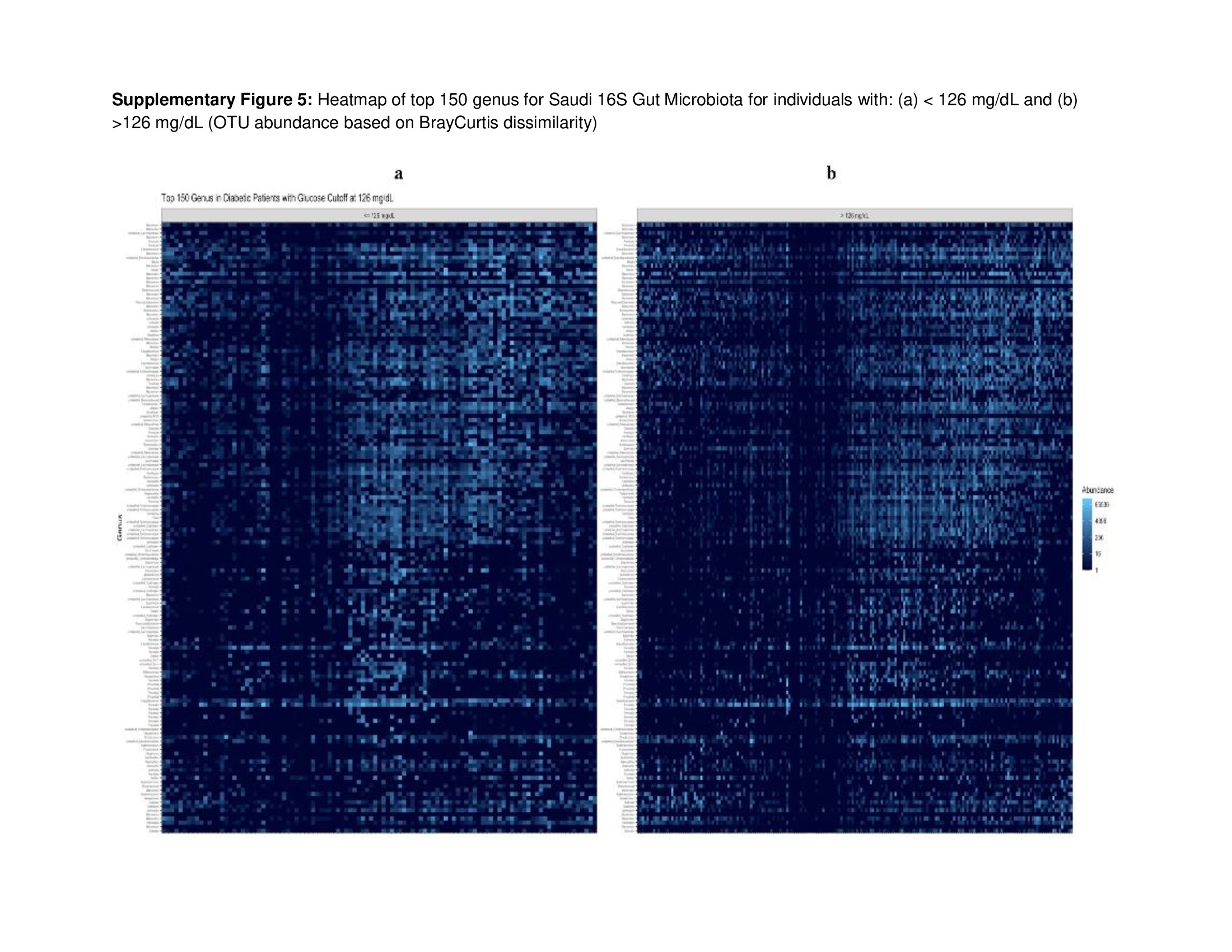

Supplement: Supplementary file 5 — Additional file 5: Supplementary Figure 5. Heatmap of top 150 genus for Saudi 16S Gut Microbiota for individuals with: (a) < 126 mg/dL and (b) > 126 mg/dL (OTU abundance based on BrayCurtis dissimilarity). [file 12866_2022_2714_MOESM5_ESM.tiff]

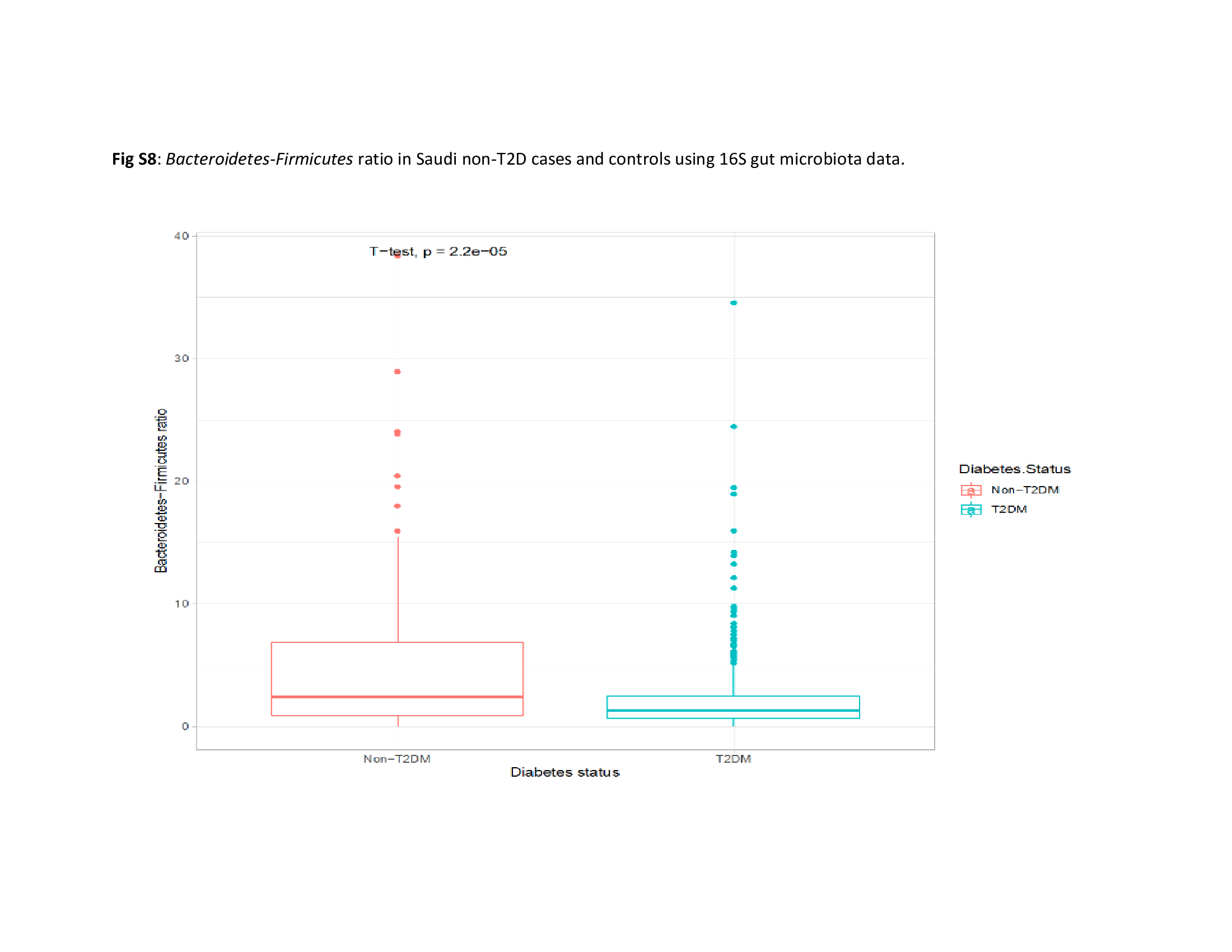

Supplement: Supplementary file 8 — Additional file 8: Fig. S8. Bacteroidetes-Firmicutes ratio in Saudi non-T2D cases and controls using 16S gut microbiota data. [file 12866_2022_2714_MOESM8_ESM.tiff]

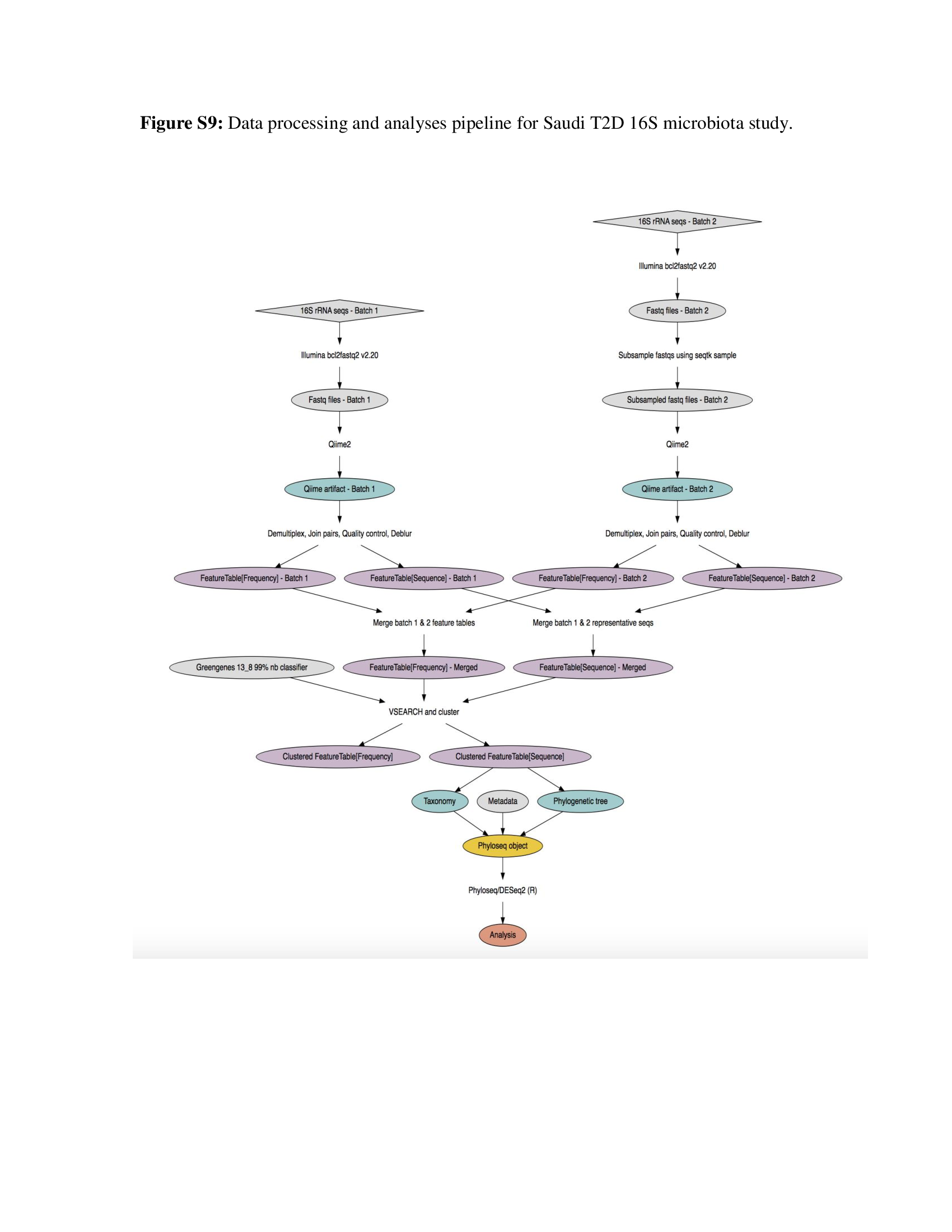

Supplement: Supplementary file 10 — Additional file 10: Figure S9. Data processing and analyses pipeline for Saudi T2D 16S microbiota study. [file 12866_2022_2714_MOESM10_ESM.tiff]
