# Supplementary material for: Gut microbiota analyses of Saudi populations for type 2 diabetes-related phenotypes reveals significant association
Source: BMC Microbiol. 2022 Dec 13;22:301. doi: 10.1186/s12866-022-02714-8 (PMC9746012; doi:10.1186/s12866-022-02714-8)
Supplement: Supplementary file 9 — Additional file 9: Supplementary Table 1. The most divergent microbiota genus between Saudi Type-2 Diabetes case and controls (Top) and between T2D cases with high (> 126 mg/ dL) and low (< 126 mg/ dL) glucose (bottom). Positive 16S fold change indicates upregulation in diabetics. [file 12866_2022_2714_MOESM9_ESM.docx]

**Supplementary Table 1:** The most divergent microbiota genus between Saudi Type-2 Diabetes case and controls (Top) and between T2D cases with high (> 126 mg/ dL) and low (< 126 mg/ dL) glucose (bottom). Positive 16S fold change indicates upregulation in diabetics.

| **Genus** | **padj** | **log2FoldChange** |
| --- | --- | --- |
| Prevotella | 5.28E-96 | -3.611 |
| unclassified_Bacteria | 2.52E-44 | 3.918 |
| Acidaminococcus | 4.50E-44 | 4.247 |
| unclassified_Bacteria | 5.65E-44 | 3.937 |
| Prevotella | 1.22E-43 | 4.030 |
| Prevotella | 6.21E-43 | -3.222 |
| Megasphaera | 8.46E-41 | 4.289 |
| unclassified_Enterobacteriaceae | 4.27E-38 | 3.984 |
| Bacteroides | 8.46E-38 | 3.737 |
| Bacteroides | 1.90E-37 | 3.965 |
| unclassified_Bacteria | 1.38E-35 | 3.327 |
| unclassified_Bacteria | 1.73E-35 | 3.393 |
| unclassified_Bacteria | 3.99E-34 | 3.205 |
| Treponema | 6.53E-34 | -1.819 |
| unclassified_Bacteria | 2.49E-30 | 3.092 |
| [Ruminococcus] | 3.76E-30 | 3.061 |
| unclassified_Enterobacteriaceae | 4.63E-29 | 3.286 |
| Dialister | 1.88E-25 | 3.156 |
| unclassified_Lachnospiraceae | 2.14E-24 | 2.793 |
| Prevotella | 8.20E-24 | 2.859 |
| Clostridium | 1.05E-23 | 2.649 |
| unclassified_Fusobacteriaceae | 1.94E-23 | -1.476 |
| Streptococcus | 1.68E-22 | 2.593 |
| Streptococcus | 1.72E-21 | 2.349 |
| unclassified_Bacteria | 2.59E-21 | 2.369 |
| Weissella | 2.69E-21 | 2.413 |
| unclassified_Bacteria | 3.22E-21 | 2.257 |
| Treponema | 1.07E-20 | -1.354 |
| Clostridium | 1.28E-20 | 2.487 |
| unclassified_Bacteroidales | 2.49E-20 | -1.669 |
| unclassified_Bacteria | 6.90E-20 | 2.326 |
| unclassified_Gammaproteobacteria | 1.83E-19 | 2.186 |
| Oscillospira | 2.12E-19 | 2.298 |
| Bacteroides | 2.43E-19 | 2.791 |
| unclassified_RF39 | 2.70E-19 | 2.387 |
| Bacteroides | 6.56E-19 | 2.682 |
| unclassified_Bacteria | 8.42E-19 | 2.105 |
| Bacteroides | 8.90E-19 | 2.334 |
| [Ruminococcus] | 1.98E-18 | 2.291 |
| unclassified_Elusimicrobiaceae | 2.82E-18 | -1.261 |
| unclassified_Bacteria | 3.10E-18 | 1.975 |
| Coprococcus | 4.89E-18 | 2.101 |
| unclassified_YS2 | 5.33E-18 | -1.395 |
| unclassified_Bacteria | 5.44E-18 | 1.976 |
| Sutterella | 9.10E-18 | 2.472 |
| Bacteroides | 1.85E-17 | 2.833 |
| Ruminococcus | 2.21E-17 | 2.365 |
| Dialister | 4.53E-17 | 2.143 |
| Faecalibacterium | 4.60E-17 | 2.408 |
| Blautia | 5.80E-17 | 2.082 |
| unclassified_Bacteroidales | 8.42E-17 | -1.165 |
| Streptococcus | 2.28E-16 | 1.897 |
| unclassified_Ruminococcaceae | 2.44E-16 | 1.962 |
| Bacteroides | 4.98E-16 | 2.238 |
| Blautia | 7.08E-16 | 1.952 |
| Veillonella | 7.98E-16 | 2.100 |
| Oscillospira | 9.53E-16 | 1.974 |
| Bacteroides | 1.02E-15 | 2.083 |
| unclassified_Lachnospiraceae | 1.06E-15 | 2.022 |
| unclassified_Bacteria | 1.41E-15 | 1.831 |
| unclassified_Comamonadaceae | 1.84E-15 | 1.983 |
| Bacteroides | 1.88E-15 | 2.313 |
| Dialister | 2.38E-15 | 2.706 |
| unclassified_Clostridiales | 2.74E-15 | 1.878 |
| unclassified_Bacteria | 2.90E-15 | 1.739 |
| Oscillospira | 3.44E-15 | 1.764 |
| Collinsella | 5.54E-15 | 1.863 |
| unclassified_Bacteria | 5.75E-15 | 1.629 |
| unclassified_Bacteria | 6.37E-15 | 1.887 |
| unclassified_Clostridiales | 6.95E-15 | -1.407 |
| unclassified_[Barnesiellaceae] | 8.37E-15 | 2.101 |
| unclassified_Bacteria | 8.99E-15 | 1.885 |
| Bacteroides | 9.34E-15 | 2.513 |
| Sutterella | 1.17E-14 | 2.029 |
| Blautia | 1.34E-14 | 1.847 |
| Bilophila | 1.75E-14 | 1.997 |
| Bacteroides | 2.17E-14 | 1.818 |
| [Prevotella] | 3.01E-14 | -1.685 |
| Ruminococcus | 4.02E-14 | 2.124 |
| Prevotella | 4.16E-14 | 2.120 |
| unclassified_Bacteria | 4.75E-14 | 1.737 |
| unclassified_Bacteria | 6.25E-14 | 1.639 |
| unclassified_Lachnospiraceae | 7.32E-14 | 1.751 |
| Parabacteroides | 7.52E-14 | 1.614 |
| Gemmiger | 8.52E-14 | 2.012 |
| Bacteroides | 9.99E-14 | 2.070 |
| Roseburia | 9.99E-14 | 2.043 |
| Paraprevotella | 1.18E-13 | 1.806 |
| unclassified_Bacteria | 1.24E-13 | 1.817 |
| Butyricimonas | 1.62E-13 | 1.760 |
| Anaerostipes | 2.29E-13 | 1.761 |
| Dorea | 2.41E-13 | 1.700 |
| unclassified_Lachnospiraceae | 2.72E-13 | 1.846 |
| Bacteroides | 3.07E-13 | 2.173 |
| Ruminococcus | 3.64E-13 | 2.076 |
| unclassified_Bacteria | 6.06E-13 | 1.655 |
| unclassified_Sphingobacteriaceae | 6.84E-13 | 1.602 |
| Unassigned | 8.55E-13 | 1.532 |
| Sutterella | 9.32E-13 | 1.903 |
| unclassified_Ruminococcaceae | 1.14E-12 | 1.860 |
| unclassified_Bacteria | 1.29E-12 | 1.448 |
| Roseburia | 2.30E-12 | 2.027 |
| unclassified_Bacteria | 2.64E-12 | 1.494 |
| Lactobacillus | 2.64E-12 | 1.741 |
| Clostridium | 2.64E-12 | 1.773 |
| unclassified_Victivallaceae | 2.87E-12 | -1.356 |
| unclassified_Erysipelotrichaceae | 3.16E-12 | 1.659 |
| unclassified_Bacteria | 4.17E-12 | 1.590 |
| Lactobacillus | 4.69E-12 | 1.582 |
| Coprococcus | 5.10E-12 | 2.128 |
| Coprococcus | 5.56E-12 | 1.546 |
| Faecalibacterium | 6.56E-12 | 1.971 |
| unclassified_Lachnospiraceae | 7.64E-12 | 1.789 |
| Lachnospira | 1.08E-11 | 2.007 |
| unclassified_Bacteria | 1.09E-11 | 1.491 |
| Pigmentiphaga | 1.56E-11 | 1.448 |
| Coprococcus | 1.86E-11 | 1.608 |
| [Ruminococcus] | 2.74E-11 | 1.514 |
| Oscillospira | 3.44E-11 | 1.481 |
| Alistipes | 3.63E-11 | 1.902 |
| unclassified_Victivallaceae | 3.72E-11 | 1.553 |
| Sutterella | 4.69E-11 | -1.070 |
| Oscillospira | 4.71E-11 | 1.317 |
| unclassified_Lachnospiraceae | 7.62E-11 | 1.456 |
| Prevotella | 7.68E-11 | -1.098 |
| unclassified_Bacteria | 9.23E-11 | 1.453 |
| unclassified_Lachnospiraceae | 9.81E-11 | 1.640 |
| unclassified_Bacteria | 1.42E-10 | 1.453 |
| Ruminococcus | 1.67E-10 | 1.483 |
| Roseburia | 2.24E-10 | 1.835 |
| unclassified_Ruminococcaceae | 2.37E-10 | 1.604 |
| Prevotella | 2.52E-10 | -1.259 |
| Oscillospira | 2.62E-10 | 1.635 |
| Clostridium | 2.62E-10 | 1.350 |
| unclassified_RF32 | 2.77E-10 | 1.414 |
| Bacteroides | 2.95E-10 | 1.650 |
| unclassified_Bacteria | 2.98E-10 | 1.306 |
| Bifidobacterium | 5.06E-10 | 1.566 |
| unclassified_Christensenellaceae | 5.60E-10 | 1.334 |
| Lachnospira | 5.62E-10 | 1.612 |
| Prevotella | 5.65E-10 | -1.324 |
| unclassified_Ruminococcaceae | 6.19E-10 | 1.431 |
| Holdemania | 7.26E-10 | 1.321 |
| unclassified_Lachnospiraceae | 8.05E-10 | 1.336 |
| unclassified_Bacteria | 8.11E-10 | 1.326 |
| unclassified_Lachnospiraceae | 8.17E-10 | 1.426 |
| Phascolarctobacterium | 1.01E-09 | 1.861 |
| [Prevotella] | 1.09E-09 | -1.842 |
| unclassified_[Mogibacteriaceae] | 1.10E-09 | 1.375 |
| Paraprevotella | 1.12E-09 | 1.751 |
| Faecalibacterium | 1.22E-09 | 1.725 |
| Prevotella | 1.26E-09 | -1.719 |
| Parabacteroides | 1.26E-09 | 1.451 |
| unclassified_Bacteria | 1.34E-09 | 1.254 |
| Faecalibacterium | 1.40E-09 | 1.643 |
| unclassified_Ruminococcaceae | 1.46E-09 | 1.352 |
| Roseburia | 1.69E-09 | 1.741 |
| unclassified_Bacteria | 2.01E-09 | 1.357 |
| unclassified_Bacteria | 2.03E-09 | 1.310 |
| unclassified_Lachnospiraceae | 2.07E-09 | 1.410 |
| unclassified_Coriobacteriaceae | 2.86E-09 | 1.466 |
| unclassified_RF32 | 3.19E-09 | 1.332 |
| unclassified_Lachnospiraceae | 3.61E-09 | 1.545 |
| unclassified_ML615J-28 | 3.65E-09 | 1.318 |
| Faecalibacterium | 3.80E-09 | 1.680 |
| unclassified_Lachnospiraceae | 4.06E-09 | 1.253 |
| unclassified_Bacteria | 4.08E-09 | 1.255 |
| Akkermansia | 4.13E-09 | 2.000 |
| unclassified_Lachnospiraceae | 4.55E-09 | 1.298 |
| Ruminococcus | 5.37E-09 | 1.370 |
| Odoribacter | 7.43E-09 | 1.470 |
| Ruminococcus | 7.68E-09 | 1.337 |
| unclassified_Bacteria | 8.82E-09 | 1.243 |
| unclassified_Bacteria | 9.58E-09 | 1.254 |
| Coprococcus | 1.04E-08 | 1.479 |
| Lachnospira | 1.13E-08 | 1.125 |
| unclassified_Christensenellaceae | 1.14E-08 | 1.251 |
| unclassified_Lachnospiraceae | 1.17E-08 | 1.133 |
| Blautia | 1.20E-08 | 1.398 |
| unclassified_Ruminococcaceae | 1.29E-08 | 1.317 |
| unclassified_Lachnospiraceae | 1.48E-08 | 1.250 |
| unclassified_[Paraprevotellaceae] | 1.58E-08 | -0.788 |
| Bacteroides | 1.96E-08 | 1.917 |
| unclassified_Christensenellaceae | 2.10E-08 | 1.145 |
| unclassified_Bacteria | 2.22E-08 | 1.148 |
| unclassified_Bacteria | 2.79E-08 | 1.146 |
| Prevotella | 3.11E-08 | 1.272 |
| Clostridium | 3.20E-08 | 1.248 |
| unclassified_[Barnesiellaceae] | 4.29E-08 | 1.141 |
| unclassified_Bacteria | 4.51E-08 | 1.172 |
| unclassified_[Barnesiellaceae] | 5.11E-08 | 1.238 |
| Oscillospira | 5.35E-08 | -0.967 |
| Roseburia | 5.76E-08 | 1.593 |
| unclassified_Bacteria | 5.96E-08 | 1.128 |
| Catenibacterium | 5.98E-08 | 1.481 |
| Paraprevotella | 6.01E-08 | 1.316 |
| unclassified_Alphaproteobacteria | 6.10E-08 | -0.765 |
| Bacteroides | 6.17E-08 | 1.743 |
| unclassified_Coriobacteriaceae | 6.19E-08 | 1.138 |
| Prevotella | 7.13E-08 | 1.359 |
| CF231 | 7.39E-08 | -1.124 |
| unclassified_Bacteria | 7.51E-08 | 1.097 |
| Clostridium | 7.51E-08 | 1.209 |
| Anaerofilum | 7.81E-08 | 1.068 |
| Dialister | 9.28E-08 | 1.499 |
| Faecalibacterium | 9.66E-08 | 1.016 |
| unclassified_S24-7 | 1.01E-07 | -1.235 |
| unclassified_Bacteria | 1.08E-07 | 1.072 |
| unclassified_Veillonellaceae | 1.11E-07 | -0.773 |
| unclassified_Bacteria | 1.12E-07 | 1.092 |
| unclassified_Clostridiales | 1.12E-07 | 1.350 |
| Coprococcus | 1.20E-07 | 1.065 |
| Sutterella | 1.28E-07 | -0.861 |
| Bacteroides | 1.32E-07 | 1.374 |
| unclassified_Clostridiales | 1.42E-07 | 1.252 |
| unclassified_Ruminococcaceae | 1.42E-07 | 1.183 |
| unclassified_Bacteria | 1.43E-07 | 1.074 |
| Alistipes | 1.46E-07 | 1.044 |
| unclassified_Bacteria | 1.55E-07 | 1.108 |
| Parabacteroides | 1.60E-07 | 1.134 |
| Ruminococcus | 1.65E-07 | 1.105 |
| Oscillospira | 1.65E-07 | 1.063 |
| Acidaminococcus | 1.79E-07 | 1.305 |
| Dorea | 1.81E-07 | 1.305 |
| Clostridium | 1.87E-07 | 0.988 |
| unclassified_Bacteria | 1.87E-07 | 1.060 |
| unclassified_Bacteria | 2.34E-07 | 1.072 |
| unclassified_Ruminococcaceae | 2.85E-07 | 1.274 |
| Ruminococcus | 3.82E-07 | 1.141 |
| unclassified_Lachnospiraceae | 4.06E-07 | 1.217 |
| Coprococcus | 4.21E-07 | 1.027 |
| Ruminococcus | 4.32E-07 | 1.128 |
| Paraprevotella | 4.64E-07 | 1.052 |
| unclassified_[Barnesiellaceae] | 4.65E-07 | -1.053 |
| Faecalibacterium | 4.74E-07 | 1.085 |
| Bacteroides | 5.13E-07 | 1.034 |
| unclassified_RF32 | 5.99E-07 | 1.228 |
| unclassified_Lachnospiraceae | 6.04E-07 | 1.393 |
| unclassified_Ruminococcaceae | 6.22E-07 | 1.172 |
| unclassified_Bacteria | 7.32E-07 | 0.953 |
| Ruminococcus | 7.39E-07 | 1.570 |
| Prevotella | 7.51E-07 | 1.052 |
| Clostridium | 7.55E-07 | 1.061 |
| Bacteroides | 7.87E-07 | 1.170 |
| Holdemania | 7.97E-07 | 1.012 |
| unclassified_Bacteria | 9.52E-07 | 1.017 |
| Coprobacillus | 9.57E-07 | 1.000 |
| unclassified_Lachnospiraceae | 9.86E-07 | 1.067 |
| Granulicatella | 1.12E-06 | 0.939 |
| Butyricimonas | 1.14E-06 | 0.998 |
| unclassified_Clostridiales | 1.26E-06 | 1.039 |
| Megamonas | 1.30E-06 | 1.384 |
| unclassified_Rikenellaceae | 1.37E-06 | 1.154 |
| Prevotella | 1.46E-06 | -0.850 |
| unclassified_Bacteria | 1.52E-06 | 0.976 |
| Phascolarctobacterium | 1.52E-06 | -0.807 |
| unclassified_Bacteria | 1.63E-06 | 1.023 |
| unclassified_Bacillales | 1.66E-06 | 1.048 |
| Sutterella | 1.91E-06 | -1.151 |
| unclassified_Bacteria | 1.92E-06 | 0.857 |
| [Ruminococcus] | 1.94E-06 | 1.138 |
| unclassified_RF32 | 1.96E-06 | 1.097 |
| unclassified_Christensenellaceae | 1.97E-06 | -0.756 |
| Bacteroides | 2.09E-06 | 1.033 |
| Lachnobacterium | 2.19E-06 | 1.258 |
| Bacteroides | 2.25E-06 | 1.062 |
| Prevotella | 2.58E-06 | -0.721 |
| unclassified_Bacteria | 2.66E-06 | 0.902 |
| unclassified_Erysipelotrichaceae | 2.75E-06 | 0.977 |
| unclassified_Bacteria | 3.33E-06 | 0.953 |
| Lactobacillus | 3.34E-06 | 0.953 |
| unclassified_Clostridiales | 4.42E-06 | 1.024 |
| Blautia | 4.51E-06 | 0.901 |
| unclassified_Ruminococcaceae | 4.59E-06 | 0.915 |
| Butyricicoccus | 4.91E-06 | 1.084 |
| Oscillospira | 4.91E-06 | 0.926 |
| unclassified_Bacteria | 5.40E-06 | 0.878 |
| unclassified_Clostridiales | 5.48E-06 | 1.087 |
| unclassified_Bacteria | 6.66E-06 | 0.875 |
| Dorea | 7.15E-06 | 0.864 |
| Oscillospira | 7.30E-06 | 0.932 |
| Coprococcus | 7.40E-06 | 1.004 |
| unclassified_Rikenellaceae | 7.43E-06 | 1.236 |
| unclassified_Christensenellaceae | 7.47E-06 | 1.244 |
| unclassified_Bacteria | 7.51E-06 | 0.827 |
| Ruminococcus | 7.66E-06 | 0.912 |
| unclassified_Bacteria | 8.02E-06 | 0.878 |
| Haemophilus | 8.02E-06 | 1.197 |
| Oscillospira | 8.15E-06 | 0.806 |
| Butyrivibrio | 8.57E-06 | -0.692 |
| Prevotella | 8.89E-06 | -1.018 |
| unclassified_Bacteria | 9.30E-06 | 0.863 |
| [Prevotella] | 9.68E-06 | 1.278 |
| Alistipes | 1.04E-05 | 1.362 |
| unclassified_Bacteria | 1.05E-05 | 0.907 |
| Bacteroides | 1.07E-05 | 0.950 |
| Lachnospira | 1.11E-05 | 1.211 |
| Paraprevotella | 1.25E-05 | 0.903 |
| unclassified_Ruminococcaceae | 1.28E-05 | 0.866 |
| Subdoligranulum | 1.29E-05 | 1.102 |
| Lactobacillus | 1.32E-05 | 0.888 |
| unclassified_Ruminococcaceae | 1.37E-05 | 1.169 |
| Oscillospira | 1.55E-05 | 0.979 |
| Parabacteroides | 1.74E-05 | 0.864 |
| Anaerotruncus | 1.93E-05 | 0.852 |
| Prevotella | 1.93E-05 | -1.383 |
| Clostridium | 1.95E-05 | 0.934 |
| Lachnospira | 1.96E-05 | 1.215 |
| unclassified_Bacteria | 1.99E-05 | 0.774 |
| unclassified_Lachnospiraceae | 2.04E-05 | 0.862 |
| unclassified_Bacteria | 2.17E-05 | 0.877 |
| unclassified_Ruminococcaceae | 2.18E-05 | 0.915 |
| unclassified_Clostridiales | 2.26E-05 | 0.868 |
| Clostridium | 2.27E-05 | 0.865 |
| Oscillospira | 2.32E-05 | 0.782 |
| unclassified_[Mogibacteriaceae] | 2.75E-05 | 0.844 |
| Slackia | 2.77E-05 | 0.879 |
| Lachnospira | 2.77E-05 | 1.191 |
| unclassified_Lachnospiraceae | 2.78E-05 | 0.856 |
| unclassified_Bacteria | 2.93E-05 | 0.839 |
| unclassified_Ruminococcaceae | 2.93E-05 | 1.170 |
| Oscillospira | 2.96E-05 | 0.846 |
| unclassified_Clostridiales | 3.05E-05 | 0.837 |
| Gemmiger | 3.12E-05 | 0.710 |
| unclassified_Lachnospiraceae | 3.14E-05 | 0.846 |
| unclassified_Bacteria | 3.14E-05 | 0.743 |
| Succinivibrio | 3.17E-05 | -1.519 |
| unclassified_Bacteria | 3.35E-05 | 0.830 |
| unclassified_Clostridiales | 3.48E-05 | -0.633 |
| unclassified_Bacteria | 3.57E-05 | 0.826 |
| Parabacteroides | 3.83E-05 | 1.307 |
| Bacteroides | 3.99E-05 | 1.341 |
| Coprococcus | 3.99E-05 | 0.838 |
| unclassified_Bacteria | 4.05E-05 | 0.772 |
| unclassified_Clostridiales | 4.07E-05 | 0.813 |
| Bacteroides | 4.07E-05 | 1.305 |
| unclassified_Bacteria | 4.16E-05 | 0.759 |
| unclassified_Bacteria | 4.34E-05 | 0.798 |
| unclassified_RF39 | 4.39E-05 | 1.053 |
| unclassified_Bacteria | 4.91E-05 | 0.768 |
| Parabacteroides | 5.03E-05 | 0.808 |
| Ruminococcus | 5.04E-05 | 0.876 |
| unclassified_Lachnospiraceae | 5.15E-05 | 0.842 |
| unclassified_RF39 | 5.25E-05 | 0.862 |
| Oscillospira | 6.03E-05 | 0.786 |
| unclassified_Lachnospiraceae | 6.15E-05 | 0.786 |
| unclassified_Ruminococcaceae | 6.15E-05 | 0.979 |
| unclassified_Bacteria | 6.18E-05 | 0.781 |
| Anaerovibrio | 6.49E-05 | -0.639 |
| Bacteroides | 6.86E-05 | 0.946 |
| unclassified_Ruminococcaceae | 6.86E-05 | -0.588 |
| Faecalibacterium | 6.92E-05 | 0.960 |
| Ruminococcus | 7.04E-05 | 0.780 |
| Oscillospira | 7.52E-05 | 1.099 |
| Unassigned | 7.60E-05 | 0.708 |
| Bacteroides | 7.76E-05 | 0.813 |
| unclassified_Clostridiales | 7.89E-05 | 0.843 |
| unclassified_Ruminococcaceae | 7.97E-05 | 0.996 |
| unclassified_Gemellaceae | 8.09E-05 | 0.735 |
| unclassified_Lachnospiraceae | 8.21E-05 | 1.003 |
| unclassified_Rikenellaceae | 8.35E-05 | 0.858 |
| unclassified_Bacteria | 8.53E-05 | 0.731 |
| unclassified_Bacteria | 9.03E-05 | 0.761 |
| unclassified_Ruminococcaceae | 9.54E-05 | 0.822 |
| Oscillospira | 0.00010086 | 0.815 |
| Pigmentiphaga | 0.00011978 | 0.768 |
| unclassified_Ruminococcaceae | 0.00012051 | 1.119 |
| unclassified_Bacteria | 0.0001233 | 0.685 |
| Oscillospira | 0.00013437 | 0.905 |
| unclassified_Bacteria | 0.0001382 | 0.761 |
| unclassified_YS2 | 0.00014108 | -0.602 |
| [Prevotella] | 0.00014465 | 0.736 |
| Ruminococcus | 0.00015272 | 0.741 |
| Lactobacillus | 0.00015298 | 0.740 |
| Bacteroides | 0.00015532 | 1.134 |
| Blautia | 0.00015748 | 0.780 |
| unclassified_Bacteria | 0.00015977 | 0.740 |
| unclassified_RF39 | 0.00016394 | 0.754 |
| Coprococcus | 0.00016394 | 0.760 |
| [Ruminococcus] | 0.00016394 | 0.707 |
| unclassified_Lachnospiraceae | 0.00016917 | 0.743 |
| Oscillospira | 0.00018929 | 0.744 |
| unclassified_Bacteria | 0.00019229 | 0.698 |
| Actinomyces | 0.0001933 | 0.668 |
| Oscillospira | 0.00019529 | 0.709 |
| unclassified_YS2 | 0.00020391 | -0.604 |
| Lactococcus | 0.00020712 | 0.719 |
| Sutterella | 0.00021375 | 0.714 |
| unclassified_Bacteria | 0.00022624 | 0.698 |
| Veillonella | 0.00023881 | 0.736 |
| Oscillospira | 0.00026391 | 0.803 |
| unclassified_Bacteria | 0.0002699 | 0.617 |
| unclassified_Lachnospiraceae | 0.00027863 | 0.728 |
| unclassified_Christensenellaceae | 0.00028121 | 0.654 |
| Lactobacillus | 0.00028677 | 0.721 |
| Ruminococcus | 0.00029444 | 0.772 |
| Oscillospira | 0.00032427 | 0.679 |
| unclassified_Bacteria | 0.00032518 | 0.649 |
| unclassified_Bacteroidetes | 0.00032518 | 0.646 |
| Bifidobacterium | 0.00032518 | 1.054 |
| Alistipes | 0.000326 | 0.776 |
| unclassified_Bacteria | 0.00032731 | 0.701 |
| Lachnospira | 0.00034118 | -0.676 |
| unclassified_Bacteroidetes | 0.00034565 | 0.661 |
| unclassified_Clostridiales | 0.00034565 | 0.734 |
| Oscillospira | 0.00035043 | -0.665 |
| Veillonella | 0.00035876 | 0.776 |
| unclassified_Clostridiales | 0.00038751 | -0.524 |
| unclassified_Clostridiales | 0.00040714 | 0.690 |
| unclassified_Coriobacteriaceae | 0.00044888 | 0.714 |
| unclassified_Coriobacteriaceae | 0.00049774 | 0.662 |
| Oscillospira | 0.00050394 | 0.728 |
| unclassified_Bacteria | 0.00051779 | 0.671 |
| unclassified_Clostridiales | 0.00052029 | 1.027 |
| unclassified_[Barnesiellaceae] | 0.00052813 | 0.853 |
| Coprococcus | 0.00052813 | -0.761 |
| unclassified_Lachnospiraceae | 0.00055752 | 0.670 |
| unclassified_Lachnospiraceae | 0.00057247 | 0.753 |
| unclassified_Christensenellaceae | 0.00057452 | 0.687 |
| Veillonella | 0.00058196 | 0.739 |
| unclassified_Bacteria | 0.00058976 | 0.663 |
| Akkermansia | 0.00058976 | 0.980 |
| unclassified_Lachnospiraceae | 0.00061214 | 0.650 |
| [Eubacterium] | 0.00063682 | 0.867 |
| Clostridium | 0.00064823 | 0.647 |
| Blautia | 0.00069914 | 0.660 |
| unclassified_Lachnospiraceae | 0.00070191 | 0.992 |
| Aggregatibacter | 0.00070706 | 0.706 |
| Ruminococcus | 0.00070755 | 0.678 |
| unclassified_Bacteria | 0.00071027 | 0.631 |
| unclassified_Lachnospiraceae | 0.00071825 | 0.736 |
| Oxalobacter | 0.00077952 | 0.679 |
| Mitsuokella | 0.00077989 | 0.837 |
| unclassified_Clostridiales | 0.00079975 | 0.687 |
| unclassified_Gammaproteobacteria | 0.00082144 | 0.642 |
| Pseudomonas | 0.00083942 | 0.645 |
| Acidaminococcus | 0.00084441 | 0.915 |
| Prevotella | 0.00084901 | 0.648 |
| unclassified_Bacteria | 0.00088442 | 0.607 |
| unclassified_Lachnospiraceae | 0.00088495 | 0.595 |
| Bacteroides | 0.00088805 | -0.789 |
| unclassified_Bacteria | 0.00090197 | 0.603 |
| [Ruminococcus] | 0.00092521 | 0.635 |
| unclassified_Ruminococcaceae | 0.00093154 | 0.967 |
| unclassified_Bacteria | 0.00094047 | 0.635 |
| Oscillospira | 0.00096276 | 0.965 |
| Ruminococcus | 0.00096547 | 0.622 |
| Ruminococcus | 0.00097253 | 0.615 |
| unclassified_Bacteria | 0.00098199 | 0.629 |
| unclassified_Coriobacteriaceae | 0.00102581 | 0.679 |
| Clostridium | 0.001028 | -0.472 |
| unclassified_Bacteroidales | 0.00103879 | -0.492 |
| unclassified_Bacteroidetes | 0.00104566 | 0.555 |
| unclassified_Bacteria | 0.00105064 | 0.591 |
| unclassified_Lachnospiraceae | 0.00106047 | 0.669 |
| Enterococcus | 0.00106681 | 0.611 |
| unclassified_Bacteria | 0.00110816 | 0.613 |
| unclassified_Bacteroidales | 0.00115877 | -0.535 |
| Oscillospira | 0.00116188 | 0.634 |
| Blautia | 0.00124093 | 0.637 |
| unclassified_Ruminococcaceae | 0.00126231 | 0.682 |
| unclassified_Clostridiales | 0.00131583 | 0.585 |
| unclassified_RF39 | 0.00131651 | 0.638 |
| Megasphaera | 0.00135276 | 0.620 |
| unclassified_Ruminococcaceae | 0.00135284 | 0.838 |
| Oscillospira | 0.00135284 | 1.069 |
| Paraprevotella | 0.00135948 | 0.721 |
| unclassified_Bacteria | 0.00149555 | 0.576 |
| Coprococcus | 0.00156719 | 0.841 |
| [Ruminococcus] | 0.00160279 | 0.590 |
| Bacteroides | 0.00161438 | 0.623 |
| unclassified_Ruminococcaceae | 0.00161438 | 0.683 |
| Butyricimonas | 0.00163939 | 0.658 |
| unclassified_Ruminococcaceae | 0.00164841 | 0.655 |
| Ruminococcus | 0.00167766 | 0.607 |
| Butyricimonas | 0.0017489 | 0.624 |
| Coprococcus | 0.00177291 | 0.573 |
| unclassified_Bacteria | 0.00186682 | 0.567 |
| unclassified_Bacteria | 0.00197866 | 0.582 |
| Clostridium | 0.0020566 | -0.542 |
| unclassified_Bacteria | 0.00206501 | 0.594 |
| unclassified_RF39 | 0.00210175 | -0.514 |
| unclassified_Clostridiales | 0.00211739 | 0.893 |
| Prevotella | 0.00218375 | 0.742 |
| Succinivibrio | 0.00219484 | 0.597 |
| [Prevotella] | 0.00224955 | -0.872 |
| unclassified_Ruminococcaceae | 0.00228652 | 0.589 |
| unclassified_Bacteria | 0.0022936 | 0.586 |
| Megasphaera | 0.00231844 | 0.602 |
| Akkermansia | 0.0023758 | 0.646 |
| Oscillospira | 0.00239137 | 0.588 |
| Alistipes | 0.00242892 | 0.853 |
| Pigmentiphaga | 0.00247398 | 0.546 |
| Ruminococcus | 0.00254367 | 0.683 |
| unclassified_Bacteria | 0.00260799 | 0.578 |
| Dorea | 0.00263114 | 0.576 |
| unclassified_Bacteria | 0.00275588 | 0.519 |
| unclassified_Bacteria | 0.00278868 | 0.557 |
| Oscillospira | 0.00280379 | 0.879 |
| unclassified_Lachnospiraceae | 0.00281076 | 0.722 |
| unclassified_Gammaproteobacteria | 0.00283636 | 0.555 |
| unclassified_Bacteria | 0.00293479 | 0.548 |
| Turicibacter | 0.00293479 | 0.582 |
| unclassified_Bacteria | 0.00298128 | 0.554 |
| Clostridium | 0.0030139 | 0.520 |
| Blautia | 0.00304179 | 0.527 |
| unclassified_Bacteria | 0.00308577 | 0.559 |
| unclassified_Bacteria | 0.00311153 | 0.559 |
| unclassified_[Barnesiellaceae] | 0.00315506 | 0.600 |
| unclassified_Lachnospiraceae | 0.00322592 | 0.520 |
| unclassified_Bacteria | 0.00325912 | 0.537 |
| unclassified_Lachnospiraceae | 0.00325912 | 0.664 |
| unclassified_Bacteria | 0.00329643 | 0.526 |
| Faecalibacterium | 0.00331368 | 0.578 |
| Veillonella | 0.00342626 | 0.579 |
| unclassified_Bacteria | 0.00351152 | 0.559 |
| unclassified_Lachnospiraceae | 0.003526 | 0.526 |
| unclassified_Enterobacteriaceae | 0.0035954 | 0.601 |
| Ruminococcus | 0.00384534 | 0.572 |
| unclassified_Clostridiales | 0.00387152 | 0.544 |
| Unassigned | 0.0038765 | 0.531 |
| unclassified_Bacteria | 0.00390843 | 0.546 |
| Rothia | 0.00392421 | 0.530 |
| Oscillospira | 0.00398582 | 0.610 |
| unclassified_Ruminococcaceae | 0.0040694 | 0.526 |
| unclassified_Clostridiales | 0.00422384 | 0.545 |
| Prevotella | 0.00448037 | 0.841 |
| Oscillospira | 0.004685 | 0.521 |
| Coprococcus | 0.00473571 | 0.563 |
| unclassified_Clostridiales | 0.00477202 | 0.563 |
| Dorea | 0.00477202 | 0.541 |
| unclassified_Bacteria | 0.00481992 | 0.542 |
| unclassified_Bacteria | 0.00483257 | 0.526 |
| unclassified_S24-7 | 0.00486528 | 0.853 |
| unclassified_Bacteria | 0.00491781 | 0.476 |
| unclassified_Clostridiales | 0.00491955 | 0.554 |
| unclassified_Lachnospiraceae | 0.00523974 | 0.533 |
| unclassified_Clostridiales | 0.00524313 | 0.531 |
| unclassified_Lachnospiraceae | 0.0052669 | 0.632 |
| Desulfovibrio | 0.00565319 | 0.576 |
| unclassified_Clostridiales | 0.00577707 | -0.542 |
| unclassified_Bacteroidetes | 0.00586415 | 0.518 |
| unclassified_Bacteria | 0.00594415 | 0.485 |
| unclassified_Bacteria | 0.00610384 | 0.493 |
| Oscillospira | 0.00624409 | 0.760 |
| unclassified_ML615J-28 | 0.00646712 | 0.625 |
| Bacteroides | 0.00654905 | 0.557 |
| unclassified_Bacteroidales | 0.00676579 | 0.578 |
| Oscillospira | 0.00676579 | 0.497 |
| Roseburia | 0.00676579 | 0.513 |
| unclassified_Bacteria | 0.00679134 | 0.482 |
| unclassified_Bacteria | 0.00685223 | 0.511 |
| nclassified_Streptophyta | 0.00710333 | 0.506 |
| unclassified_Bacteria | 0.00716854 | 0.511 |
| unclassified_[Barnesiellaceae] | 0.00735597 | 0.578 |
| unclassified_Bacteria | 0.00737146 | 0.492 |
| unclassified_Bacteria | 0.00751562 | 0.516 |
| unclassified_RF39 | 0.00764707 | 0.496 |
| unclassified_Bacteria | 0.00764827 | 0.448 |
| Actinomyces | 0.00764827 | 0.466 |
| unclassified_Bacteria | 0.00796176 | 0.513 |
| Dehalobacterium | 0.00816457 | 0.491 |
| unclassified_Ruminococcaceae | 0.00816457 | 0.490 |
| unclassified_[Mogibacteriaceae] | 0.00833368 | 0.582 |
| Alistipes | 0.00837117 | 0.632 |
| Butyricimonas | 0.0084985 | 0.502 |
| unclassified_Lachnospiraceae | 0.00854386 | 0.482 |
| unclassified_Bacteria | 0.00859651 | 0.491 |
| unclassified_Bacteria | 0.00867263 | 0.478 |
| Clostridium | 0.00867263 | 0.498 |
| Parabacteroides | 0.00868438 | 0.517 |
| unclassified_Bacteria | 0.00885099 | 0.487 |
| Butyricimonas | 0.00891743 | 0.486 |
| Bifidobacterium | 0.0090374 | 0.489 |
| unclassified_Bacteria | 0.00926061 | 0.469 |
| unclassified_Ruminococcaceae | 0.00945952 | 0.486 |
| unclassified_Lachnospiraceae | 0.00951284 | 0.484 |
| unclassified_Christensenellaceae | 0.00956124 | 0.460 |
| Oribacterium | 0.00956124 | -0.381 |
| unclassified_Bacteria | 0.00958349 | 0.437 |
| unclassified_Bacteria | 0.0095848 | 0.471 |
| Unassigned | 0.00966028 | 0.429 |
| Dialister | 0.00982296 | 0.592 |
| Streptococcus | 0.00983538 | 0.437 |
| Ruminococcus | 0.00991988 | 0.482 |
